# Comparative Proteomics Profiling Illuminates the Fruitlet Abscission Mechanism of Sweet Cherry as Induced by Embryo Abortion

**DOI:** 10.3390/ijms21041200

**Published:** 2020-02-11

**Authors:** Zhi-Lang Qiu, Zhuang Wen, Kun Yang, Tian Tian, Guang Qiao, Yi Hong, Xiao-Peng Wen

**Affiliations:** 1 Key Laboratory of Plant Resources Conservation and Germplasm Innovation in Mountainous Region (Guizhou University), Ministry of Education, Institute of Agro-bioengineering/College of Life Sciences, Guizhou University, Guiyang 550025, China; 18786621377@163.com (Z.-L.Q.); gzu_zwen@163.com (Z.W.); kyanggz@163.com (K.Y.); 13518504594@163.com (G.Q.); hongyi715@163.com (Y.H.); 2 Institute for Forest Resources & Environment of Guizhou, College of Forestry, Guizhou University, Guiyang 550025, China; tiantiangzu@163.com

**Keywords:** sweet cherry, embryo abortion, fruitlet abscission, fruit drop, mechanism

## Abstract

Sweet cherry (*Prunus avium* L.) is a delicious nutrient-rich fruit widely cultivated in countries such as China, America, Chile, and Italy. However, the yield often drops severely due to the frequently-abnormal fruitlet abscission, and few studies on the metabolism during its ripening process at the proteomic level have been executed so far. To get a better understanding regarding the sweet cherry abscission mechanism, proteomic analysis between the abscising carpopodium and non-abscising carpopodium of sweet cherry was accomplished using a newly developed Liquid chromatography-mass spectrometry/mass spectrometry with Tandem Mass Tag (TMT-LC-MS/MS) methodology. The embryo viability experiments showed that the vigor of the abscission embryos was significantly lower than that of retention embryo. The activity of cell wall degrading enzymes in abscising carpopodium was significantly higher than that in non-abscising carpopodium. The anatomy results suggested that cells in the abscission zone were small and separated. In total, 6280 proteins were identified, among which 5681 were quantified. It has been observed that differentially accumulated proteins (DAPs) influenced several biological functions and various subcellular localizations. The Kyoto Encyclopedia of Genes and Genomes (KEGG) enrichment analysis showed that plenty of metabolic pathways were notably enriched, particularly those involved in phytohormone biosynthesis, cell wall metabolism, and cytoskeletal metabolism, including 1-aminocyclopropane-1-carboxylate oxidase proteins which promote ethylene synthesis, and proteins promoting cell wall degradation, such as endoglucanases, pectinase, and polygalacturonase. Differential expression of proteins concerning phytohormone biosynthesis might activate the shedding regulation signals. Up-regulation of several cell wall degradation-related proteins possibly regulated the shedding of plant organs. Variations of the phytohormone biosynthesis and cell wall degradation-related proteins were explored during the abscission process. Furthermore, changes in cytoskeleton-associated proteins might contribute to the abscission of carpopodium. The current work represented the first study using comparative proteomics between abscising carpopodium and non-abscising carpopodium. These results indicated that embryo abortion might lead to phytohormone synthesis disorder, which effected signal transduction pathways, and hereby controlled genes involved in cell wall degradation and then caused the abscission of fruitlet. Overall, our data may give an intrinsic explanation of the variations in metabolism during the abscission of carpopodium.

## 1. Introduction

Sweet cherry (*Prunus avium* L.), widely cultivated in countries such as China, America, and Japan, is an important fruit crop known for its appealing color, delicious taste, and nutritional value [1]. However, abnormal fruit abscission can often reduce crop yield greatly. Previous research shows that fruitlet shedding is insufficient to obtain better economic benefits [2]. Fruit abscission is a highly regulated developmental process is effected by both internal and environmental causes [3]. Its regulatory mechanism is complicated and concerns multiple reasons. Therefore, efforts on the unveiling molecular mechanism of fruit abscission in sweet cherry plays vital role in increasing its yield.

Abscission is a fundamental process in plant biology and represents an evolutionary adaptation of plants, it allows to discard senescent or physiologically damaged organs, e.g., leaves, petals, and fruit [4,5] for better adaptation and for efficient seed dispersal [6]. Abscission is precisely regulated by structural, physiological, biochemical, and molecular changes that ultimately lead to the shedding of plant organs [7]. This event takes place in a special cell layer called as abscission zone (AZ), which consists of cell separation enabled by hydrolytic enzymes [5,8]. However, frequently severe abscission is a hard nut for fruit productivity [9]. Recently, more efforts have been leveraged on illuminating the regulation of abnormal abscission at the molecular level [10,11,12], which help understand the mechanisms underlying abscission along with getting bumper harvest [4,6]. Also, molecular studies on abscission can help improve current agricultural management practices, such as flower and fruit thinning, mechanical picking of fruit [4,11]. To date, the abundant studies on organ abscission had been described in the model plants, e.g., *Arabidopsis thaliana* [13] and *Solanum lycopersicum* [12,14,15], and some molecular knowledge related to fruit abscission had also been acquired from fruit tree crops (e.g., apple, citrus, lichi) [6,11,16]. However, the current information about the molecular mechanisms underlying severe fruit abscission in sweet cherry has not yet been unraveled.

According to the causes, abscission can be divided into three types, namely, normal abscission (such as abscission of ripened fruit and seed), metabolic abscission due to the completion between the reproductive growth and vegetative growth (such as premature shedding of fruit and unpollinated flowers), and abnormal abscission owing to environmental stresses (such as cold, heat, light, and pathogen) [17]. The abscission of plant organs is associated with a balance between the levels of auxin and ethylene in AZ [18,19,20]. It has also been observed that ethylene can induce the synthesis and secretion of various cell wall and middle hydrolases, which are accompanying to plant organ abscission [9,21], while auxin inhibits abscission by rendering AZ cells insensitive to ethylene [10,12]. Simply, abscission can be divided into four major steps: (a) Differentiation and formation of the AZ; (b) acquisition of the competence to respond to abscission signals; (c) execution of organ abscission; and (d) differentiation of a protective layer [8,14]. It has been found that the expression of multiple regulatory genes varied before and during peduncle shedding [22], and this variation affected the differential expression of transcription factors associated with the auxin and ethylene pathways [23]. 

Typical components of the cell wall containing cellulose, hemicellulose, pectic polysaccharides, proteins, and phenolic compounds. During the process of plant organ shedding, cell wall hydrolases are synthesized in large quantities, and enzyme activity is also elevated, which conceivably the origin of the degradation of the middle lamella and the loosening of the primary cell wall of the separation layers [21]. Cellulase (CEL) and polygalacturonase (PG), two major cell wall hydrolase enzymes, had been extensively studied in different plants and played an important role in plant organ abscission [24]. Additionally, expansin protein (EXP), xyloglucan endotransglucosylases/hydrolases (XTH), peroxidase (POD) [15,25] also play an essential function during plant organ abscission. In the process of ethylene induction or low level auxin initiation, the degrading enzyme genes of plant cell walls were also up-regulated, resulting in the abscission of plant organs [26]. 

Comparative proteomic analysis is a powerful tool for systematically understanding of a biological event at the molecular level [14]. Recently, proteomics has been widely used in the study of citrus [27], apple [28], pear [29] and sweet cherry [30], etc. However, there had not been reports on the mechanism of cherry fruit drop by proteomic analysis. Besides, Parallel Reaction Monitoring (PRM) was a recently developed methodology in targeted mass spectrometry, which involves in the use of a quadrupole-equipped orbitrap [31] and has been widely used to quantify and detect target proteins [32,33]. Also, PRM has been used to validate the reliability of proteomic data, which provides a reliable guarantee for accurate and reliable proteomics data [34]. 

In the present study, the vigor of abscission sweet cherry embryos was inspected, the enzyme activity assay of the abscising acropodium and non-abscising carpopodium, the anatomical structure observations of the abscising acropodium and non-abscising carpopodium, then we used TMT and PRM to analyze the changes in the proteome during the embryo-induced abortion. The study found that (1) the degree of embryo abortion was significantly higher in normal fruits than in normal fruits; (2) the cell wall degrading enzyme activity was significantly higher than retention fruit; (3) the cells in the abscission zone were small and separated; (4) A total of 6280 proteins were identified, among which 5681 were quantified. A total of 1957 DAPs, including 1056 up- and 901 down-regulated proteins, were identified. Mostly, preceding proteins involved in cell wall hydrolysis-related, lignin synthesis-related, plant hormone synthesis and signaling-related enzymes, cytoskeleton-related proteins, transporters, and transcription factors. Existing study can provide data for scrutinizing the shedding of plant organs from the aspects of morphology, anatomy and molecular biology, also establishes a foundation revealing the molecular mechanism of sweet cherry fruit abscission and breeding high yield sweet cherry germplasm.

## 2. Results

### 2.1. Embryo Vigor of Shedding Fruit

**Figure 1:**
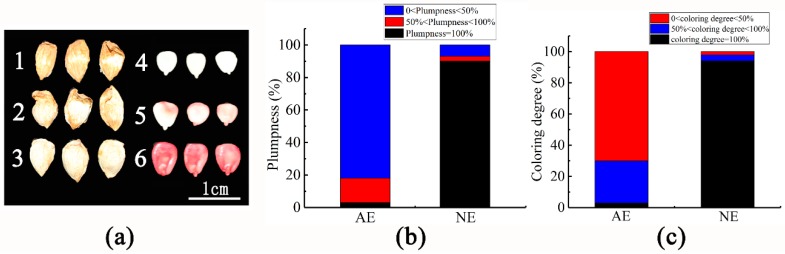
The embryos vigor of abscission and non-abscission fruit. (**a**) 1, 2, and 3 represent different plumpness, and 4, 5, and 6 represent the coloring degree corresponding to different plumpness. (**b**), The stacked figure of different plumpness. (**c**), The stacked figure of different coloring degree. AE, abscission embryo; NE, non-abscission embryo.

After anatomical observation of embryos during the development, they might be divided into three types according to the plumpness, namely 0 < plumpness < 50% (Figure 1(a1)), 50% < plumpness < 100% (Figure 1(a2)) and plumpness = 100% (Figure 1(a3)); according to the statistics, it is shown that in the abscission fruit, the fruit with plumpness = 100% accounts for 3%, while in the normal fruit, the fruit with plumpness = 100% accounts for only 90% (Figure 1b). Based on the results of the staining experiment, it can be divided into three categories, namely 0 < coloring degree < 50% (Figure 1(a4)), 50% < coloring degree < 100% (Figure 1(a5)) and coloring degree = 100% (Figure 1(a6)). In the abscission fruits, coloring degree = 100% accounted for 3%, while the non-abscission fruits accounted for 94% (Figure 1c). The result showed that the fruit plumpness and the coloring degree of embryos are related to fruit abscission. In other words, there was a greater correlation between embryo abortion and fruit shedding.

### 2.2. Enzyme Activity and Anatomical Structure of Carpopodium Abscission Zone

**Figure 2:**
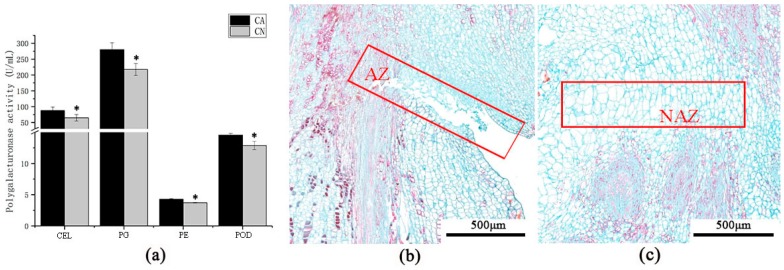
Enzyme activity and anatomical structure of carpopodium abscission zone. (**a**) The activities of cellulase (CEL), polygalacturonase (PG), pectinase (PE), and peroxidase (POD) in the abscising carpopodium (CA) and non-abscising carpopodium (CN); (**b**) The anatomical structure of abscising carpopodium abscission zone. (**c**) The anatomical structure of the non-abscising carpopodium abscission zone. AZ, abscission zone. NAZ, non-abscising abscission zone.

To explore whether cell wall hydrolysis and activity of antioxidant activity-related enzymes peroxidase in the abscission zone is related to the shedding of sweet cherry fruitlet. Enzyme activity assay showed that cellulase (CEL), polygalacturonase (PG), pectinase (PE), and peroxidase (POD) activities were significantly higher than retention carpopodium (Figure 2a). This result indicates that the shedding of sweet cherry fruit may be due to the hydrolysis of the cell wall in the abscission zone. In addition, the anatomical structure of carpopodium abscission zone suggested that abscising carpopodium abscission zone cell was small and dense (Figure 2b), while the non-abscising carpopodium abscission zone cell was sizeable and lean (Figure 2c). Moreover, the cells in the abscission zone (AZ) also separated. These outcomes indicate that the fruit abscission of the sweet cherry has an immense connection with the physiological, biochemical metabolism and cell structure of the abscission zone.

### 2.3. Quality Control and Quantitative Proteomic Analysis

**Figure 3:**
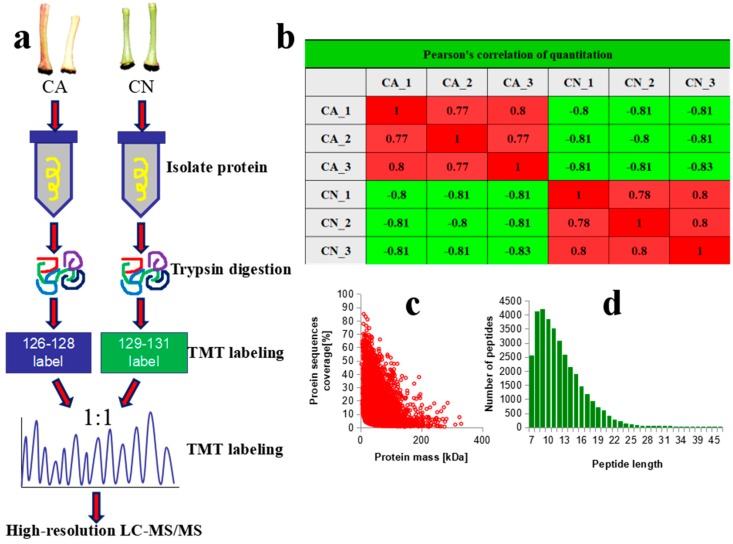
The trial technique for quantitative proteome investigation and quality control approval of MS information. (**a**) Protein was extricated in three natural imitates for each sample gathering. Entire protein samples were trypsin digested and dissected by HPLC-MS/MS. (**b**) Pearson s correlation of protein quantitation. (**c**) Mass delta of all identified peptides. (**d**) Length distribution of every single distinguished peptide.

An integrated approach involving LC-MS/MS and TMT labeling was applied to analyze the proteomic changes between abscising carpopodium and non-abscising carpopodium. The general workflow is demonstrated in Figure 3a. The satisfactory reproducibility for the current experiment has been proven via Pair-wise Pearson’s correlation coefficients (Figure 3b). Overall, 34,432 peptides were revealed. Following the quality confirmation, along with average mass error < 0.02 Da, signifying an immense validity for data regarding MS (Figure 3c). Classified peptides lengths were recorded among 7 to 20 amino acids, which demonstrating fulfilled standard criteria of our sampling (Figure 3d). The number of proteins analyzed during the experiment was 6280, where 5681 were quantified. All discovered proteins were categorized to understand their function properly e.g., GO terms, represent the functional domains, KEGG pathways, and subcellular localization. The identified protein’s detailed information is listed in Table S1.

### 2.4. Identification of DAPs During Carpopodium Abscission

A total of 1957 DAPs, including 1056 up- and 901 down-regulated proteins, were recognized (Table S2 and Figure S1). Among DAPs, the top five up-regulated proteins were a 7-deoxyloganetin glucosyltransferase-like (4.39 fold), followed by a plasma membrane-associated cation-binding protein 1 (3.70), an UDP-glycosyltransferase 73C1-like (3.66 fold), a putative Beta-D-xylosidase (3.59 fold), an extracellular ribonuclease LE-like (3.40 fold). the top five down-regulated proteins were a glucuronoxylan 4-*O*-methyltransferase 1 (4.81 fold), anthocyanidin reductase ((2*S*)-flavan-3-ol-forming, 4.00 fold), probable auxin efflux carrier component 1c (3.86 fold), cytochrome P450 98A2 (3.57 fold), dCTP pyrophosphatase 1-like (3.40 fold) (Table S2). Subcellular locations of the DAPs were predicted (Table S3). For the up-regulated proteins, a total of 14 groups were identified, such as chloroplast- (404 proteins), cytoplasm- (201), nucleus- (168), plasma membrane- (99), extracellular- (82), vacuolar membrane- (42), mitochondria- (30), and endoplasmic reticulum- (12 proteins) (Figure S1). For the down-regulated proteins, 15 components were identified, including chloroplast- (309 proteins), cytoplasm- (267), nucleus- (189), and plasma membrane-localized protein (58) (Figure S1).

### 2.5. Enrichment Analysis of DAPs During Carpopodium Abscission

**Figure 4:**
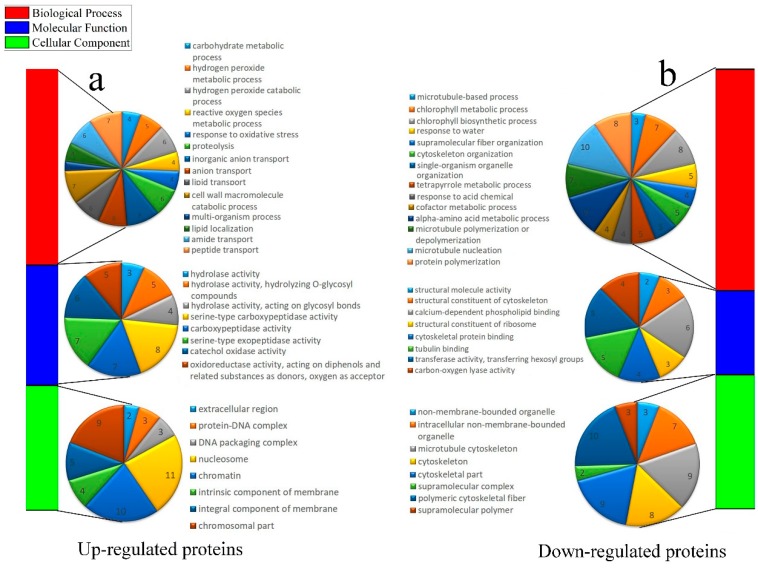
Gene Ontology (GO) enrichment analysis of DAPs. (**a**) Dispersion of the up-regulated proteins with GO annotation. Diverse shading squares express to various terms, including cellular component, molecular function, and biological process. The number, some of the up-regulated proteins in each second-level term, was appeared in a pie chart. (**b**) Distribution of the down-regulated proteins with GO annotation. Diverse colors squares symbolize to various terms, including cellular component, molecular function, and biological process. Number, the quantity of the down-regulated proteins in each second-level term has appeared in a pie chat.

In total, 332 DAPs were assigned to at least one GO term. For up-regulated proteins, the highly enriched ‘Biological Process’ GO terms were ‘inorganic anion transport’, ‘cell wall macromolecule catabolic process’, ‘peptide transport’; within the ‘Molecular Function’, the most significantly enriched terms were ‘hydrolase activity’; and the most enriched terms in the ‘Cellular Component’ were ‘nucleosome’ and ‘chromatin’ (Figure 4a). For down-regulated proteins, the highly enriched ‘Biological Process’ GO terms were related to ‘microtubule nucleation’, ‘alpha-amino acid metabolic process’, ‘chlorophyll biosynthetic process’, and ‘protein polymerization’. Within the ‘Molecular Function’, the most significantly enriched terms were ‘structural molecule activity’, ‘structural constituent of cytoskeleton’; and the most enriched terms in the ‘Cellular Component’ were ‘polymeric cytoskeletal fiber’, ‘microtubule cytoskeleton’, ‘cytoskeletal part’, and ‘cytoskeleton’ (Figure 4b).

The up-regulated proteins were mostly linked with ‘phenylpropanoid biosynthesis’ and ‘galactose metabolism’; and the down-regulated proteins were usually engaged in ‘flavonoid biosynthesis’ and ‘amino acids’ (Figure S2). 

The up-regulated proteins generally contained a Glycoside hydrolase superfamily and down-regulated proteins mainly comprised a TCP-1-like chaperonin intermediate domain (Figure S3).

### 2.6. Biosynthesis of Cell Wall Modifying Proteins and Lignin

**Figure 5:**
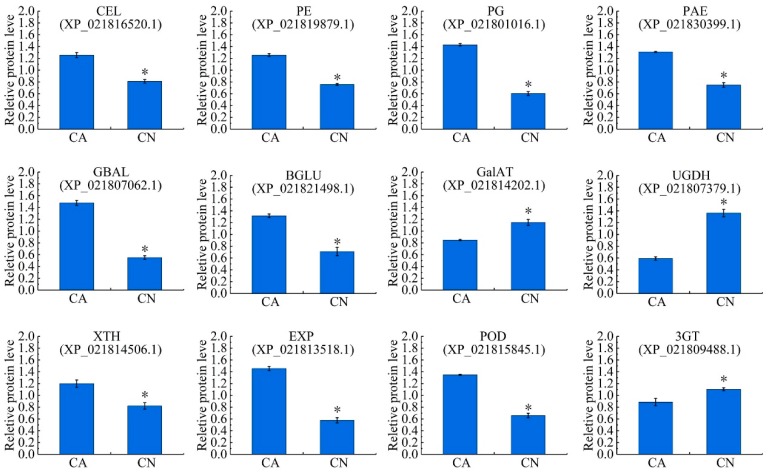
Relative expression levels of the proteins related to cell wall metabolism and lignin biosynthesis. Significant differences in expression level were indicated by “*”.

Among all the DAPs, 101 proteins were predicted to be associated with cell wall metabolism and lignin biosynthesis, of which 73 were up-regulated and 28 down-regulated in the abscising carpopodium (Table S4). These included, cell wall hydrolytic enzymes such as endoglucanase CX (CEL), pectinesterase (PE), polygalacturonase (PG), pectin acetylesterase 12-like (PAE), β-galactosidase (GBAL), beta-glucosidase 45-like isoform X6 (BGLU) significantly up-regulated in the abscising carpopodium; however, the biosynthesis of cell wall modifying proteins, namely, galacturonosyltransferase (GalAT) and UDP-glucose 6-dehydrogenase (UGDH) were significantly down-regulated in the abscising carpopodium. Interestingly, the extension-associated proteins cell wall, e.g., xyloglucan endotransglucosylase/hydrolase protein (XTH) and expansin-like B1 (EXP), were significantly up-regulated in the abscising carpopodium. Excluding, the related proteins of lignin synthesis, e.g., peroxidase 16-like (POD), were also significantly up-regulated, while anthocyanidin 3-*O*-glucosyltransferase 5-like (3GT) significantly down-regulated in abscising carpopodium (Table S4, Figure 5). 

### 2.7. Plant Hormone Biosynthesis and Signal Transduction

**Figure 6:**
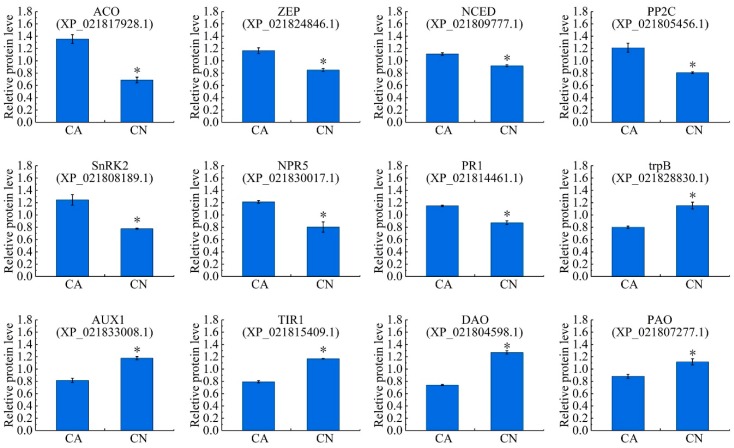
Relative expression levels of the proteins related to plant hormone and signal transduction. Significant differences in expression level were indicated by “*”.

Totally, 105 proteins were annotated to be associated with the phytohormone biosynthesis and signal transduction pathways, of which 51 were up-regulated and 54 down-regulated. In the ethylene biosynthesis pathway, four 1-aminocyclopropane-1-carboxylate oxidase (ACO) proteins were significantly up-regulated in the abscising carpopodium. In the abscisic acid biosynthesis pathway, the zeaxanthin epoxidase (ZEP) and 9-cis-epoxycarotenoid dioxygenase (NCED) were significantly up-regulated in the abscising carpopodium. In the abscisic acid signal transduction pathway, ten protein phosphatase 2C (PP2C) proteins and one SnRK2 were significantly up-regulated. In the salicylic acid signal transduction pathway, one NPR5 and one pathogenesis-related protein 1 (PR1) were significantly up-regulated. In the auxin biosynthesis pathway, two tryptophan synthase alpha chain, the enzyme necessary for the synthesis of tryptophan was significantly down-regulated in the abscising carpopodium. In the auxin signal transduction pathway, the auxin transporter-like protein 2 (AUX1) and transport inhibitor response 1 (TIR1) were significantly down-regulated in abscising carpopodium. furthermore, one 2-oxoglutarate-dependent dioxygenase (DAO), which is essential for auxin catabolism and the maintenance of auxin homeostasis in reproductive organs was down-regulated in abscising carpopodium. It is worth mentioning that one polyamine oxidase was down-regulated in abscising carpopodium (Table S5, Figure 6). 

### 2.8. Cytoskeleton and Transport Proteins

Totally, 49 proteins were related with the cytoskeleton, of which 23 were up-regulated and 26 were down-regulated. These proteins mainly concerned tubulin family proteins, microtubule-associated proteins, and actin regulated proteins. These proteins mainly involved in the formation of cell wall. Therefore, these proteins may regulate the detachment of the carpopodium by regulating the formation of cell walls. Among the transport proteins, of which 27 were up-regulated and 14 were down-regulated prior proteins holding ABC transporters, lipid-transfer proteins, calcium-transporting ATPase, auxin transport protein, etc. These proteins also regulate cell wall hydrolase changes by transporting ATP, lipids, calcium, and auxins.

### 2.9. Transcription Factor

Totally, 17 transcription factors were accumulated, involved different family TFs including homeobox-leucine zipper, bZIP and ARF, bHLH, suggesting a complex regulation of organ separation. In particular, homeobox-leucine zipper protein ATHB-12-like was up-regulated 2.62 fold, bZIP was up-regulated 1.21 fold, bHLH3 was down-regulated 1.30 fold and ARF was down-regulated 1.38 fold, which may play a critical role in the abscission of plant organs.

### 2.10. Validation of DAPs by PRM

**Figure 7:**
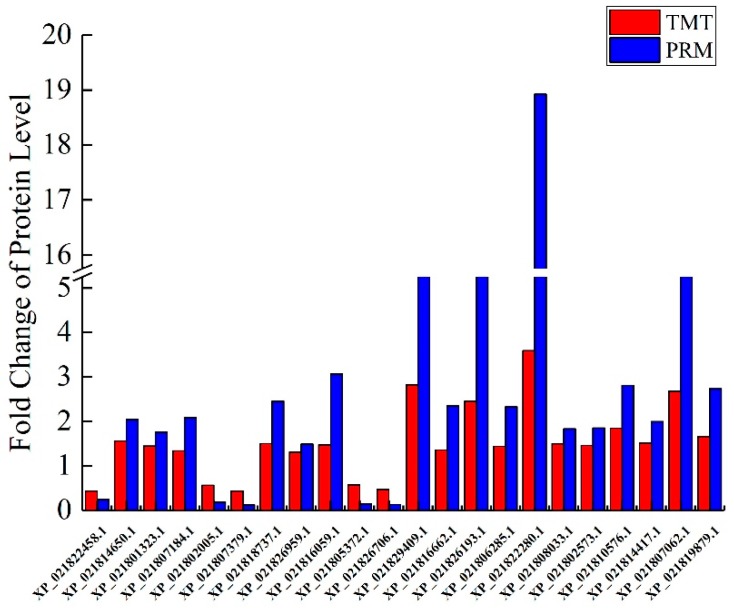
Confirmation of 22 selected differentially produced proteins detected by the Parallel Reaction Monitoring (PRM) technique. The ordinate represents the CA/CN ratio value, ratio > 1 represents the up-regulation, and ratio < 1 represents the down-regulation.

Totally, 22 DAPs significantly involved in these GO terms and pathways were selected for PRM analysis. Additionally, two particular peptides along anticipated chemical stability were selected of separate protein, and the relative protein abundance was indicated as the average of the two standardized peptide top regions (Table S6). The expression values of the up-regulated proteins were higher and those of the down-regulated proteins were lower in the CA group, in comparison to the CN group. The fold changes for these proteins were significantly different between the CA and CN groups at *p* < 0.01, in agreement with the findings from TMT analysis (Figure 7).

## 3. Discussion

Fruit abscission cause by embryo abortion presents in a variety of plants, such as apple [35] and mango [36]. However, there are few studies on embryo abortion-induced sweet cherry fruit shedding. It was not until the early 21st century that people began to study the physiological mechanism of sweet cherry fruit abscission, and these studies have shown that the fruit abscission has an excellent at correlation with the polar transport of auxin, carbohydrate, and abscisic acid [37,38]. Recently, a large number of evidences on fruit shedding have been published about other species, such as tomato [23], citrus [6], and litchi [16]. It is noteworthy that high-throughput proteomic analysis has been developed to reveal the mechanism of plant organ abscission at the protein level [14]. Abscission is a process that works together by external and internal factors [3]. The process has a complex mechanism or modification processes [14], including cell wall modifications [6], plant hormonal biosynthesis, signal transduction pathways [3], and pathogen defense-regulated [4]. To study the fruitlet abscission mechanism of sweet cherry as induced by embryo abortion, comparative proteomics was used to study alterations in protein between the abscising carpopodium and non-abscising carpopodium. 

### 3.1. Embryo Abortion Leads to Fruit Abscission

In the present case, experiments from embryo vigor and fruitlet abscission demonstrated that there was a positive correlation between embryo vigor and fruitlet abscission. It can be inferred that the abortion of an embryo may cause fruit shedding. Both mango [36] and citrus [39] have proved that embryo abortion can lead to fruit abscission. Additionally, our group also found that the pollination trees have low pollen vigor and pollen deformity under an electron microscope (Unpublished). In the process of fruit setting and development, endogenous hormones play a continuous coordinating role, and embryo abortion is related to the content and balance of endogenous hormones [40]. In turn, numerous studies have found that the regulation of endogenous hormones is closely related to the embryonic development of plants [41]; in other words, the plant hormone imbalance can also cause embryo abortion. There were many reasons for embryo abortion, such as male sterility [42], pollination and fertilization [43], and endogenous hormones [41]. Therefore, by adjusting the hormone balance and improving the pollen vigor of pollination tree, the abortion rate of the embryo can be reduced and the fruit setting rate can be increased. If not, after finding the molecular mechanism of fruit shedding, it can also increase fruit yield by directly regulating the expression of genes associated with fruit shedding.

### 3.2. Cell Wall Metabolism and Abscission

Abscission is an active physiological process that occurs through the dissolution of cell walls at predetermined locations, the abscission zones (AZs) [3]. The most direct cause of plant organ is due to changes in cell wall hydrolase activity, resulting in the degradation of the cell wall. Several genes regulate the functioning of the plant cell wall. Changes in their expression are associated with aging [5], organ growth and development [5,44], maturation of fruits [45], and organ abscission [46].

Our proteomics profiling of sweet cherry abscising carpopodium showed bidirectional changes in the expression of the cell wall-related proteins, probably associated not only with the ongoing process of abscission but also with the progressive development of the organs that are not dropped (Table S4). However, it is worth noting that most of the enzymes involved in cell wall degradation showed a trend of up-regulation, including cellulose [3], pectinase [47], polygalacturonase [48], β-galactosidase [49], which play a major role in cell wall degradation. Moreover, the enzymatic activity of cellulose, pectinase and polygalacturonase were significantly higher than non-abscising carpopodium (Figure 2a). Besides, expansin was proven to be associated with the process of wall extension during cell growth [15]. It has, however, become clear that expansins also make a significant contribution to the process of fruit softening, which involves wall breakdown, rather than expansion. It has been observed that expansins play an important role with ethylene-mediated abscission. The function of expansin may increase the disorder of cellulose crystals, making the glucan chains easier to hydrolyze [50]. In the current case, significant accumulation of the expansin in the abscising carpopodium, the accumulation of expansin may play an important role in the shedding of sweet cherry carpopodium. Xyloglucan is one of the major hemicelluloses of primary cell walls, in dicot plants, and may account for up to 10%–20% of cell wall components [15]. XTHs belong to a multigene family which plays important role in several different processes during cell wall modification. These include root hair initiation [51], hypocotyl elongation [52], hydrolysis of seed storage carbohydrates, leaf growth and expansion, fruit softening tension, wood formation, and petal abscission. Currently, two XTHs were significantly up-regulated in abscising carpopodium (Figure 6), suggesting that It is likely that changes mediated by XTHs action may allow easier accessibility of the cell wall to other cell wall hydrolytic enzymes, thus accelerating abscission [32].

### 3.3. Lignin Biosynthesis and Abscission

Despite the fact that the role of lignin and lignified tissues in abscission has not yet been explained, it had been notable that during leaf abscission of woody species, ligno-suberization of the protective layers is exceptionally normal [9]. Moreover, in the process of plant organ shedding, it was usually accompanied by lignin deposition [6]. The function of lignin discharge has been related to the generation of defensive layers at the tissues staying in the plant during the last step of the abscission procedure [9,53]. Moreover, it has been suggested that lignification could also facilitate the mechanical cell wall breakage during cell separation processes [54]. Therefore, lignin deposition might be considered as a marker of abscission activation. In the present study, there were several proteins significantly up-regulated in ‘Phenylpropanoid biosynthesis’ pathway. It is noteworthy that 7 peroxidases were up-regulated over 1.5 times. Additionally, the enzymatic activity of peroxidases was significantly higher than the non-abscising carpopodium (Figure 2a), suggesting an increase in lignin biosynthesis. This result indicated that ‘peroxidase’ plays an important role in the process of lignin biosynthesis. However, lignin plays an important role in the shedding of carpopodium and the formation of protective layers.

### 3.4. Plant Hormone-Related Abscission

In the overall process of abscission, regulatory effects of plant hormones are of major relevance since they mediate responses of plant organs to stress [4,55]. Depending on their concentration in different tissues, their receptor concentration and affinity, their homeostasis, their transport or their interaction, hormones can act as a signal to accelerate or inhibit the effects of abscission, and the responses are complex [3]. The ethylene and abscisic acid (ABA) act as abscission-accelerating signals [3,18], while auxin was considered as abscission inhibitors [13,56]. The plant hormone ethylene plays an important role as a positive regulator in process of plant organ abscission since it can induce differential gene expression, including cell wall hydrolytic enzymes, lipid-transfer proteins, pathogen-related proteins, hormone biosynthesis, etc. [9]. However, it remains unclear whether ABA induces abscission directly via hormone activity or indirectly by generating a mild carbohydrate deficit [57]. Auxin inhibits the plant organ abscission because it can prevent the expression of some cell wall degrading enzymes [13]. Before auxin worked, it was transported by the auxin influx carriers (AUX1/LAX) and transport inhibitor response 1 (TIR1) [58]. Therefore, AUX1 and TIR1 also play an important role in plant organ abscission. Recently, four key proteins for ethylene biosynthesis, 1-aminocyclopropane-1-carboxylate oxidase (ACO) proteins, were up-regulated significantly in the abscising carpopodium. However, the S-adenosylmethionine synthetase (SAMS) were down-regulated. The down regulation of SAMS could increase methionine accumulation [59], while the up-regulated of ACO could increase ethylene biosynthesis [60]. These results indicate that ethylene biosynthesis increases lead to the carpopodium abscission. In the ‘carotenoid biosynthesis’ pathway, the ABA biosynthesis key protein 9-cis-epoxycarotenoid dioxygenase (NCED) was up-regulated significantly in the abscising carpopodium. In addition, in the ABA signal transduction pathway, one PP2C and two SnRK2 was significantly up-regulated, the evidence suggests that abscisic acid may play an important role in the carpopodium abscission. It is noteworthy that tryptophan synthase was down-regulated in the abscising carpopodium. This result led to the decrease in tryptophan synthesis, thereby causing a decrease in the synthesis of auxin. Additionally, the AUX1 and TIR1 protein down-regulated in the abscising carpopodium. These results indicate the precursor of auxin synthesis and the transport vector of auxin are reduced, resulting in a decrease in auxin entering the cell. In summary, the synthesis of ethylene and abscisic acid-related proteins were significantly up-regulated, while the synthesis of auxin-related proteins was significantly down-regulated, leading to imbalance of hormones, leading to the shedding of the carpopodium.

### 3.5. Cytoskeleton and Abscission

The cytoskeleton is a fundamental component of the constituent cells, including the actin cytoskeleton and the microtubule cytoskeleton. Studies have shown that adhesion maintenance of requires modification of the cell wall, depending on the actin cytoskeleton [61]. The reason why is that the delivery of pectin and its modifying proteins occur mainly via the actin cytoskeleton. To regulate branching and nucleation of the actin filament, the Actin-associated protein2/3 complex (Arp2/3) is highly conserved and is the critical component [62]. In addition, the microtubule cytoskeleton is also the basic structure of cellulose. There have been reports that there is a great correlation between the arrangement of microtubules and shedding [63]. Besides, microtubule-associated proteins have an important role in cellulose biosynthesis [64]. Currently, one actin-related protein 2/3 complex is significantly down-regulated in the abscising carpopodium. This result indicates that the ability to adhere to cells is limited, which increases the possibility of shedding. On the other hand, a total of seven microtubule-associated proteins are significantly down-regulated. These results indicate that the synthesis of microtubules has been inhibited. These results indicate that cell wall synthesis decreases, leading to decreased adhesion between cells, causing cell separation (Figure 2b) and carpopodium abscission.

### 3.6. Transcription Factor Raleted Abscission

During the plant organ abscission, a large number of transcription factors are also involved. Including ERF family [49], ARF family [13], MADS-box family [65,66], and HD-ZIP family [16,67]. In the present study, ERF and MADS-box did not accumulate, However, two HD-ZIP family proteins were significantly up-regulated. In litchi, two cellulase genes, *LcCEL2* and *LcCEL8*, can be directly activated by the HD-ZIP family transcription factor HB2, synthesize cellulase, and then hydrolyze cellulose, causing the loss of litchi fruits [67]. Besides, when the fruitlets sense the abscission signal, LcHB2/3 is induced, which stimulates the biosynthesis of ethylene and ABA through direct binding to the promoters of *LcACO2/3*, *LcACS1/4/7*, and *LcNCED3* genes., fruitlets sense the abscission signals. Afterward, *LcPG1/2* gene expression and polygalacturonase action are expanded. Additionally, ethylene and ABA may also boost the expression of LcHB2/3 by positive feedback regulation. The final breakdown of homogalacturonan in the cell walls of FAZ leads to the occurrence of fruitlet abscission [16].

## 4. Materials and Methods 

### 4.1. Plant Materials

The sweet cherry ‘Santina’, grown in Weining County, Guizhou Province, China (E: 104.12, N: 27.25) was used as the material, and the abscising carpopodium and the non-abscising carpopodium were taken during the young fruit period. Three plants with similar growth vigor were selected, and abscising carpopodium (CA) and non-abscising carpopodium (CN) were taken at 20 d after flowering. These carpopodiums were quickly frozen in liquid nitrogen and brought back to the laboratory for storage in a −80 °C refrigerator. Also, to explore the relationship between fruit shedding and embryo development, embryos of abscission and retention fruits were used to detect vitality.

### 4.2. Detection of Embryo Activity

In this study, 100 retention fruits and 100 abscission fruits were randomly selected, dissected, and the appearance was observed. According to the size of the embryo, we define the percentage of embryos in the seed coat called plumpness. Afterward, embryo activity was examined. By 2,3,5-Triphenyte-trazoliumchloride (TTC). According to how much the embryo is colored, the percentage of the embryo colored as coloring degree.

### 4.3. Measurement of Enzyme Activity 

The abscising carpopodium (CA) and non-abscising carpopodium (CN) were taken, and their activities of cellulase, pectinase, peroxidase and polygalacturonase were determined by the kit (solabio, Beijing, China) according to the description, and each measurement index was repeated three times.

### 4.4. Anatomical Observation of Carpopodium Abscission Zones.

The carpopodium of ‘Santina’retention and abscission fruit were sampled, then the optimized glycerol alcohol mixture (50% glycerol: 70% alcohol in a volume ratio of 1:1) was used to soften carpopodium for at least 24 h. Subsequently, cross sections containing fruit stalk abscission layer with 1–2 cm in length were taken from the fruit, and were fixed immediately in FAA (Formalin-acetic acid-alcohol, 70%) for at least 48 h. Then, pretreated carpopodium were dehydrated in an ethanol series (70%, 85%, 95% (*v*/*v*)) and absolute ethanol, embedded in paraffin, sectioned at a thickness of 8–10 μm cross-sections by a rotary microtome. and dyed with safranin-fast green and slice was sealed by Canada balsam. All prepared slides were observed with a CX41RF light microscope (Olympus, Tokyo, Japan). Photographs were taken with a digital camera. Digital images in JPEG format were again processed with Photoshop CS6 (Adobe Systems Incorporated, San Jose, CA, US).

### 4.5. Protein Extraction and Trypsin Digestion

The extraction of plant proteins was performed based on the published methods with a slight modification. Briefly, the sample was ground with liquid N_2_ into cell powder and transferred to a 5-mL tube. Next the sample was sonicated three times on ice using a high intensity ultrasonic processor (Scientz) in four volumes of lysis buffer (8 M urea, 10 mM dithiothreitol, 1% Protease Inhibitor Cocktail). The remaining debris was removed by centrifugation at 20,000 g at 4 °C for 10 min. Finally, the protein was precipitated with cold 20% TCA for 2 h at −20 °C. After centrifugation at 12,000 *g* 4 °C for 10 min, the supernatant was discarded. The remaining precipitate was washed with cold acetone for three times. The protein was redissolved in 8 M urea and the protein concentration was determined with BCA kit according to the manufacturer’s instructions.

For digestion, the protein solution was reduced with 5 mM dithiothreitol for 30 min at 56 °C and alkylated with 11 mM iodoacetamide for 15 min at room temperature in darkness. The protein sample was later diluted by adding 100 mM TEAB to urea concentration < 2M. Finally, trypsin was added into the protein sample in a 1:50 trypsin-to-protein mass ratio for the first digestion overnight, and 1:100 trypsin-to-protein mass ratio for a second 4 h-digestion.

### 4.6. TMT Labeling and HPLC Fractionation

After trypsin digestion, sample peptides were desalted by Strata X C18 SPE column (Phenomenex, Torrance, CA, US) and vacuum-dried. Resulting peptides were reconstituted using a six-plex TMT kit (ThermoFisher, Shanghai, China) according to its operation manual. Briefly, one unit of TMT reagent was thawed and reconstituted in acetonitrile. The peptides mixtures were then incubated for 2 h at room temperature and pooled, desalted and dried by vacuum centrifugation. 

The sample peptides were fractionated into fractions by high pH reverse-phase HPLC using the Agilent 300Extend C18 column (Agilent, Shanghai, China). Briefly, sample peptides were fractionated with a gradient of 8%–32% acetonitrile (pH 9.0) over 60 min into 60 fractions. Then, all fractions were combined into 18 fractions and vacuum dried by centrifuging.

### 4.7. LC-MS/MS Analysis

Peptides were dissolved in 0.1% formic acid and directly loaded onto a homemade reversed-phase analytical column (15-cm length, 75 μm i.d.). The gradient was comprised of an increase from 6% to 23% solvent B (0.1% formic acid in 98% acetonitrile) over 26 min, 23% to 35% in 8 min and climbing to 80% in 3 min then holding at 80% for the last 3 min, all at a constant flow rate of 400 nL/min on an EASY-nLC 1000 UPLC system (Thermo, Shanghai, China).

The peptides were subjected to NSI source followed by tandem mass spectrometry (MS/MS) in Q ExactiveTM Plus (Thermo, Shanghai, China) coupled online to the UPLC. The electrospray voltage applied was 2.0 kV. The m/z scan range was 350 to 1800 for full scan, and intact peptides were detected in the Orbitrap at a resolution of 70,000. Peptides were then selected for MS/MS using the NCE setting as 28 and the fragments were detected in the Orbitrap at a resolution of 17,500. A data-dependent procedure that alternated between one MS scan followed by 20 MS/MS scans with 15.0s dynamic exclusion. Automatic gain control (AGC) was set at 5E4. The fixed first mass was set as 100 *m*/*z*.

### 4.8. Database Search

The resulting MS/MS data were processed using the Maxquant with integrated Andromeda search engine v.1.5.2.8 (Matthias Mann Lab, Germany). Tandem mass spectra were searched against a *Prunus avium* database concatenated with reverse decoy database. Trypsin/P was specified as cleavage enzyme allowing up to 2 missing cleavages. The mass tolerance for precursor ions was set as 20 ppm in the first search and 5 ppm in the main search, and the mass tolerance for fragment ions was set as 0.02 Da. Carbamidomethyl on Cys was specified as fixed modification and oxidation on Met was set as variable modifications. False discovery rate (FDR) was adjusted to < 1% and minimum score for peptides was set > 40 A TMT-6-plex kit was used for quantification of the resulting peptides. The quantitative level of the peptide was measured according to its ion signal intensity ratio in the secondary spectrum.

### 4.9. Bioinformatics Analysis

The Gene Ontology (GO) annotation proteome was derived from the UniProt-GOA database (http://www.ebi.ac.uk/GOA/), first converting the identified protein ID to a UniProt ID and then mapping to GO IDs by protein ID. If some identified proteins are not annotated by UniProt-GOA database, the InterProScan soft are used to annotated protein’s GO functional based on protein sequence alignment method. GO items can be divided into three categories, namely, biological process (BP), cellular component (CC), molecular function (MF). In this study, we mapped the differentially displayed proteins (fold changes > 1.2, *p* < 0.05) into the GO database (http://www.geneontology.org/). It was computable for the amount of proteins at each GO term and the target list used for results, which came from TMT data. The list was constructed by downloading the data on the GO database. The Kyoto Encyclopedia of Genes and Genomes (KEGG) database (https://www.kegg.jp/) was used to annotate the protein pathway, first using KEGG online service tools KAAS to annotate the protein s KEGG database description and then mapping the annotation result on the KEGG pathway database using KEGG online service tools KEGG mapper.

## 5. Conclusions

**Figure 8:**
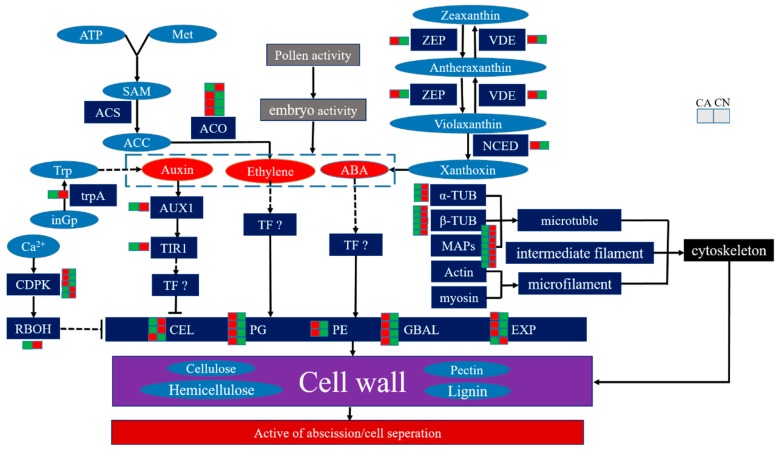
Model of abscission regulation. Red ovals represent hormones, dark blue represents enzymes or proteins, light blue ellipse represents compounds or ions, small red boxes represent up-regulated proteins, and small green squares represent down-regulated proteins.

In this study, through protein sequencing and related physiological indicators, it was found that the mechanism of sweet cherry fruit drop may be caused by pollen abortion. Pollen abortion caused embryo abortion, then it might lead to phytohormone synthesis disorder, which effected signal transduction pathways, and hereby controlled genes involved in cell wall degradation and then caused the abscission of fruitlet. Overall, our data may give an intrinsic explanation of the variations in metabolism during the abscission of carpopodium (Figure 8).

## Supplementary Materials

Supplementary materials can be found at https://www.mdpi.com/1422-0067/21/4/1200/s1.

## Abbreviations

3GT	anthocyanidin 3-*O*-glucosyltransferase 5-like
ABA	abscisic acid
ACO	1-aminocyclopropane-1-carboxylate oxidase
AE	Abscission embryo
AGC	Automatic gain control
ARF	Auxin response factor
Arp2/3	Actin-related protein2/3 complex
AUX1	auxin transporter-like protein 2
AZ	Abscission Zone
BGLU	beta-glucosidase
bHLH	basic-helix-loop-helix
BP	biological process
bZIP	basic region/leucine zipper motif
CA	abscising carpopodium
CC	cellular component
CEL	cellulase
CN	non-abscising carpopodium
DAO	2-oxoglutarate-dependent dioxygenase DAO-like
DAPs	Differential accumulated proteins
EXP	Expansin
FAA	Formalin-acetic acid-alcohol
FDR	False discovery rate
GalAT	galacturonosyltransferase
GBAL	β-galactosidase
GO	gene ontology
HPLC	High-Performance Liquid Chromatography
KEGG	The Kyoto Encyclopedia of Genes and Genomes
MF	molecular function
NE	Non-abscission embryo
NPR5	regulatory protein NPR5-like
PAE	pectin acetylesterase
PE	pectinesterase
PG	polygalacturonase
POD	peroxidase
PP2C	protein phosphatase 2C
PR1	pathogenesis-related protein 1
PRM	Parallel Reaction Monitoring
PRM	Parallel Reaction Monitoring
SAMS	S-adenosylmethionine synthetase
SnRK2	SNF1-related protein kinase 2
TIR1	transport inhibitor response 1
TMT	Tandem Mass Tag
TMT-LC/MS	Tandem Mass Tag-liquid chromatograph-mass spectrometer
TTC	Triphenyte-trazoliumchloride
UGDH	UDP-glucose 6-dehydrogenase
XTH	xyloglucan endotransglucosylase/hydrolase protein
ZEF	zeaxanthin epoxidase

## References

[1] Wei H., Chen X., Zong X., Shu H., Gao D., Liu Q. (2015). Comparative transcriptome analysis of genes involved in anthocyanin biosynthesis in the red and yellow fruits of sweet cherry (*Prunus avium* L.). PLoS ONE.

[2] Kühn N., Serrano A., Abello C., Arce A., Espinoza C., Gouthu S., Deluc L., Arce-Johnson P. (2016). Regulation of polar auxin transport in grapevine fruitlets (*Vitis vinifera* L.) and the proposed role of auxin homeostasis during fruit abscission. BMC Plant Biol..

[3] Sawicki M., Aït Barka E., Clément C., Vaillant-Gaveau N., Jacquard C. (2015). Cross-talk between environmental stresses and plant metabolism during reproductive organ abscission. J. Exp. Bot..

[4] Estornell L.H., Agustí J., Merelo P., Talón M., Tadeo F.R. (2013). Elucidating mechanisms underlying organ abscission. Plant Sci..

[5] Glazinska P., Wojciechowski W., Kulasek M., Glinkowski W., Marciniak K., Klajn N., Kesy J., Kopcewicz J. (2017). De novo transcriptome profiling of flowers, flower pedicels and pods of lupinus luteus (*Yellow lupine*) reveals complex expression changes during organ abscission. Front. Plant Sci..

[6] Merelo P., Agustí J., Arbona V., Costa M.L., Estornell L.H., Gómez-Cadenas A., Coimbra S., Gómez M.D., Pérez-Amador M.A., Domingo C. (2017). Cell wall remodeling in abscission zone cells during ethylene-promoted fruit abscission in citrus. Front. Plant Sci..

[7] Parra-Lobato M.C., Gomez-Jimenez M.C. (2011). Polyamine-induced modulation of genes involved in ethylene biosynthesis and signalling pathways and nitric oxide production during olive mature fruit abscission. J. Exp. Bot..

[8] Patterson S.E. (2001). Cutting loose. Abscission and dehiscence in Arabidopsis. Plant Physiol..

[9] Agustí J., Merelo P., Cercós M., Tadeo F.R., Talón M. (2008). Ethylene-induced differential gene expression during abscission of citrus leaves. J. Exp. Bot..

[10] Nakano T., Fujisawa M., Shima Y., Ito Y. (2013). Expression profiling of tomato pre-abscission pedicels provides insights into abscission zone properties including competence to respond to abscission signals. BMC Plant Biol..

[11] Nakano T., Kato H., Shima Y., Ito Y. (2015). Apple SVP family MADS-box proteins and the tomato pedicel abscission zone regulator JOINTLESS have similar molecular activities. Plant Cell Physiol..

[12] Fu X., Shi Z., Jiang Y., Jiang L., Qi M., Xu T., Li T. (2019). A family of auxin conjugate hydrolases from Solanum lycopersicum and analysis of their roles in flower pedicel abscission. BMC Plant Biol..

[13] Ellis C.M., Nagpal P., Young J.C., Hagen G., Guilfoyle T.J., Reed J.W. (2005). AUXIN RESPONSE FACTOR1 and AUXIN RESPONSE FACTOR2 regulate senescence and floral organ abscission in *Arabidopsis thaliana*. Development.

[14] Zhang X.L., Qi M.F., Xu T., Lu X.J., Li T.L. (2015). Proteomics profiling of ethylene-induced tomato flower pedicel abscission. J. Proteom..

[15] Tsuchiya M., Satoh S., Iwai H. (2015). Distribution of XTH, expansin, and secondary-wall-related CesA in floral and fruit abscission zones during fruit development in tomato (*Solanum lycopersicum*). Front. Plant Sci..

[16] Li C., Ma X., Huang X., Wang H., Wu H., Zhao M., Li J. (2019). Involvement of HD-ZIP I transcription factors LcHB2 and LcHB3 in fruitlet abscission by promoting transcription of genes related to the biosynthesis of ethylene and ABA in litchi. Tree Physiol..

[17] Qi M.F., Xu T., Chen W.Z., Li T.L. (2014). Ultrastructural localization of polygalacturonase in ethylene-stimulated abscission of tomato pedicel explants. Sci. World J..

[18] Taylor J.E., Whitelaw C.A. (2001). Signals in abscission. New Phytol..

[19] Meir S., Hunter D.A., Chen J., Halaly V., Reid M.S. (2006). Molecular Changes Occurring during Acquisition of Abscission Competence following Auxin Depletion in *Mirabilis jalapa*. Plant Physiol..

[20] Sundaresan S., Philosoph-Hadas S., Riov J., Belausov E., Kochanek B., Tucker M.L., Meir S. (2015). Abscission of flowers and floral organs is closely associated with alkalization of the cytosol in abscission zone cells. J. Exp. Bot..

[21] Mishra A., Khare S., Trivedi P.K., Nath P. (2008). Effect of ethylene, 1-MCP, ABA and IAA on break strength, cellulase and polygalacturonase activities during cotton leaf abscission. South African J. Bot..

[22] Gao Y., Liu C., Li X., Xu H., Liang Y., Ma N., Fei Z., Gao J., Jiang C.Z., Ma C. (2016). Transcriptome profiling of petal abscission zone and functional analysis of an Aux/IAA family gene RhiAA16 involved in petal shedding in rose. Front. Plant Sci..

[23] Sundaresan S., Philosoph-Hadas S., Riov J., Mugasimangalam R., Kuravadi N.A., Kochanek B., Salim S., Tucker M.L., Meir S. (2016). De novo transcriptome sequencing and development of abscission zone-specific microarray as a new molecular tool for analysis of tomato organ abscission. Front. Plant Sci..

[24] Roberts J.A., Gonzalez-Carranza Z.H. (2009). Pectinase functions in abscission. Stewart Postharvest Rev..

[25] Poovaiah B.W., Rasmussen H.P. (1973). Calcium Distribution in the Abscission Zone of Bean Leaves. Plant Physiol..

[26] Patterson S.E., Bleecker A.B. (2004). Ethylene-Dependent and -Independent Processes Associated with Floral Organ Abscission in Arabidopsis. Plant Physiol..

[27] Wang J.H., Liu J.J., Chen K.L., Li H.W., He J., Guan B., He L. (2017). Comparative transcriptome and proteome profiling of two Citrus sinensis cultivars during fruit development and ripening. BMC Genom..

[28] Zhang S., Zhang D., Fan S., Du L., Shen Y., Xing L., Li Y., Ma J., Han M. (2016). Effect of exogenous GA3 and its inhibitor paclobutrazol on floral formation, endogenous hormones, and flowering-associated genes in ‘Fuji’ apple (*Malus domestica* Borkh.). Plant Physiol. Biochem..

[29] Li J.M., Huang X.S., Li L.T., Zheng D.M., Xue C., Zhang S.L., Wu J. (2015). Proteome analysis of pear reveals key genes associated with fruit development and quality. Planta.

[30] Chan Z., Wang Q., Xu X., Meng X., Qin G., Li B., Tian S. (2008). Functions of defense-related proteins and dehydrogenases in resistance response induced by salicylic acid in sweet cherry fruits at different maturity stages. Proteomics.

[31] Bargiela R., Herbst F.A., Martínez-Martínez M., Seifert J., Rojo D., Cappello S., Genovese M., Crisafi F., Denaro R., Chernikova T.N. (2015). Metaproteomics and metabolomics analyses of chronically petroleum-polluted sites reveal the importance of general anaerobic processes uncoupled with degradation. Proteomics.

[32] Tsuchiya H., Tanaka K., Saeki Y. (2013). The parallel reaction monitoring method contributes to a highly sensitive polyubiquitin chain quantification. Biochem. Biophys. Res. Commun..

[33] Yu Q., Liu B., Ruan D., Niu C., Shen J., Ni M., Cong W., Lu X., Jin L. (2014). A novel targeted proteomics method for identification and relative quantitation of difference in nitration degree of OGDH between healthy and diabetic mouse. Proteomics.

[34] Dong H., Li Y., Fan H., Zhou D., Li H. (2019). Quantitative proteomics analysis reveals resistance differences of banana cultivar ‘Brazilian’ to Fusarium oxysporum f. sp. cubense races 1 and 4. J. Proteom..

[35] Ferrero S., Carretero-Paulet L., Mendes M.A., Botton A., Eccher G., Masiero S., Colombo L. (2015). Transcriptomic signatures in seeds of apple (*Malus domestica L*. Borkh) during fruitlet abscission. PLoS ONE.

[36] He J.H., Ma F.W., Chen Y.Y., Shu H.R. (2012). Differentially expressed genes implicated in embryo abortion of mango identified by suppression subtractive hybridization. Genet. Mol. Res..

[37] Blanusa T., Else M.A., Atkinson C.J., Davies W.J. (2005). The regulation of sweet cherry fruit abscission by polar auxin transport. Plant Growth Regul..

[38] Blanusa T., Else M.A., Davies W.J., Atkinson C.J. (2006). Regulation of sweet cherry fruit abscission: The role of photo-assimilation, sugars and abscisic acid. J. Horticultural Sci. Biotechnol..

[39] Mesejo C., Muñoz-Fambuena N., Reig C., Martínez-Fuentes A., Agustí M. (2014). Cell division interference in newly fertilized ovules induces stenospermocarpy in cross-pollinated citrus fruit. Plant Sci..

[40] Ben-Cheikh W., Perez-Botella J., Tadeo F.R., Talon M., Primo-Millo E. (1997). Pollination increases gibberellin levels in developing ovaries of seeded varieties of citrus. Plant Physiol..

[41] Tokuji Y., Kuriyama K. (2003). Involvement of gibberellin and cytokinin in the formation of embryogenic cell clumps in carrot (*Daucus carota*). J. Plant Physiol..

[42] Pellan-Delourme R., Renard M. (1988). Cytoplasmic male sterility in rapeseed (*Brassica napus* L.): Female fertility of restored rapeseed with “Ogura” and cybrids cytoplasms. Genome.

[43] Lal N., Kumar Gupta A., Nath V. (2017). Fruit Retention in Different Litchi Germplasm Influenced by Temperature. Int. J. Current Microbiol. Appl. Sci..

[44] Gunawardena A.H.L.A.N., Greenwood J.S., Dengler N.G. (2007). Cell wall degradation and modification during programmed cell death in lace plant, Aponogeton madagascariensis (Aponogetonaceae). Amer. J. Bot..

[45] Giné-Bordonaba J., Echeverria G., Ubach D., Aguiló-Aguayo I., López M.L., Larrigaudière C. (2017). Biochemical and physiological changes during fruit development and ripening of two sweet cherry varieties with different levels of cracking tolerance. Plant Physiol. Biochem..

[46] Kim J., Sundaresan S., Philosoph-Hadas S., Yang R., Meir S., Tucker M.L. (2015). Examination of the abscission-associated transcriptomes for soybean, tomato, and arabidopsis highlights the conserved biosynthesis of an extensible extracellular matrix and boundary layer. Front. Plant Sci..

[47] Goldental-Cohen S., Burstein C., Biton I., Ben Sasson S., Sadeh A., Many Y., Doron-Faigenboim A., Zemach H., Mugira Y., Schneider D. (2017). Ethephon induced oxidative stress in the olive leaf abscission zone enables development of a selective abscission compound. BMC Plant Biol..

[48] Ke X., Wang H., Li Y., Zhu B., Zang Y., He Y., Cao J., Zhu Z., Yu Y. (2018). Genome-wide identification and analysis of polygalacturonase genes in solanum lycopersicum. Int. J. Mol. Sci..

[49] Gao Y., Liu Y., Liang Y., Lu J., Jiang C., Fei Z., Jiang C.Z., Ma C., Gao J. (2019). Rosa hybrida RhERF1 and RhERF4 mediate ethylene- and auxin-regulated petal abscission by influencing pectin degradation. Plant J..

[50] Cosgrove D.J. (1999). Enzymes and Other Agents That Enhance Cell Wall Extensibility. Annu. Rev. Plant Physiol. Plant Mol. Biol..

[51] Vissenberg K., Fry S.C., Verbelen J.P. (2001). Root hair initiation is coupled to a highly localized increase of xyloglucan endotransglycosylase action in arabidopsis roots. Plant Physiol..

[52] Carmen C., Jocelyn R.K.C., Alan B.B. (1997). Auxin regulation and spatial localization of an endo-1,4-beta-D-glucanase and a xyloglucan endotransglycosylase in expanding tomato hypocotyls. Plant J..

[53] Van Nocker S. (2009). Development of the abscission zone. Stewart Postharvest Rev..

[54] Liljegren S.J., Ditta G.S., Eshed Y., Savidge B., Bowmant J.L., Yanofsky M.F. (2000). SHATTERPROOF MADS-box genes control dispersal in Arabidopsis. Nature.

[55] Smékalová V., Doskočilová A., Komis G., Šamaj J. (2014). Crosstalk between secondary messengers, hormones and MAPK modules during abiotic stress signalling in plants. Biotechnol. Adv..

[56] Nakano T., Ito Y. (2013). Molecular mechanisms controlling plant organ abscission. Plant Biotechnol..

[57] Einhorn T.C., Arrington M. (2018). ABA and Shading Induce ‘Bartlett’ Pear Abscission and Inhibit Photosynthesis but Are Not Additive. J. Plant Growth Regul..

[58] Shen C.J., Bai Y.H., Wang S.K., Zhang S.N., Wu Y.R., Chen M., Jiang D.A., Qi Y.H. (2010). Expression profile of PIN, AUX/LAX and PGP auxin transporter gene families in Sorghum bicolor under phytohormone and abiotic stress. FEBS J..

[59] Goto D.B., Ogi M., Kijima F., Kumagai T., Van Werven F., Onouchi H., Naito S. (2002). A single-nucleotide mutation in a gene encoding S-adenosylmethionine synthetase is associated with methionine over-accumulation phenotype in Arabidopsis thaliana. Genes Genet. Syst..

[60] Chersicola M., Kladnik A., Žnidarič M.T., Mrak T., Gruden K., Dermastia M. (2017). 1-Aminocyclopropane-1-Carboxylate Oxidase Induction in Tomato Flower Pedicel Phloem and Abscission Related Processes Are Differentially Sensitive To Ethylene. Front. Plant Sci..

[61] Daher F.B., Braybrook S.A. (2015). How to let go: Pectin and plant cell adhesion. Front. Plant Sci..

[62] Higgs H.N., Pollard T.D. (2001). Regulation of Actin Filament Network Formation Through ARP2/3 Complex: Activation by a Diverse Array of Proteins. Annual Rev. Biochem..

[63] Li Y., Du M., Tian X., Xu D., Li Z. (2016). Study on the changes of micr otubule cytoskeleton of abscission zone dur ing leaf abscission in cotton (in chinese). J. Shihezi Univ..

[64] Rajangam A.S., Kumar M., Aspeborg H., Guerriero G., Arvestad L., Pansri P., Brown C.J.L., Hober S., Blomqvist K., Divne C. (2008). MAP20, a microtubule-associated protein in the secondary cell walls of hybrid aspen, is a target of the cellulose synthesis inhibitor 2,6-dichlorobenzonitrile. Plant Physiol..

[65] Wing R.A., Mao L., Begum D., Chuang H., Budiman M.A., Szymkowiak E.J., Irish E.E. (2000). JOINTLESS is a MADS-box gene controlling tomato flower abscission zone development. Nature.

[66] Xie Q., Hu Z., Zhu Z., Dong T., Zhao Z., Cui B., Chen G. (2014). Overexpression of a novel MADS-box gene SlFYFL delays senescence, fruit ripening and abscission in tomato. Sci. Rep..

[67] Li C., Zhao M., Ma X., Wen Z., Ying P., Peng M., Ning X., Xia R., Wu H., Li J. (2019). The HD-Zip transcription factor LcHB2 regulates litchi fruit abscission through the activation of two cellulase genes. J. Exp. Bot..

