# Supplementary material for: Comparative Proteomics Profiling Illuminates the Fruitlet Abscission Mechanism of Sweet Cherry as Induced by Embryo Abortion
Source: Int J Mol Sci. 2020 Feb 11;21(4):1200. doi: 10.3390/ijms21041200 (PMC7072775; doi:10.3390/ijms21041200)
Supplement: Supplementary file 1 [file ijms-21-01200-s001.zip › Supplementary Files-IJMS/Figure S3.docx]

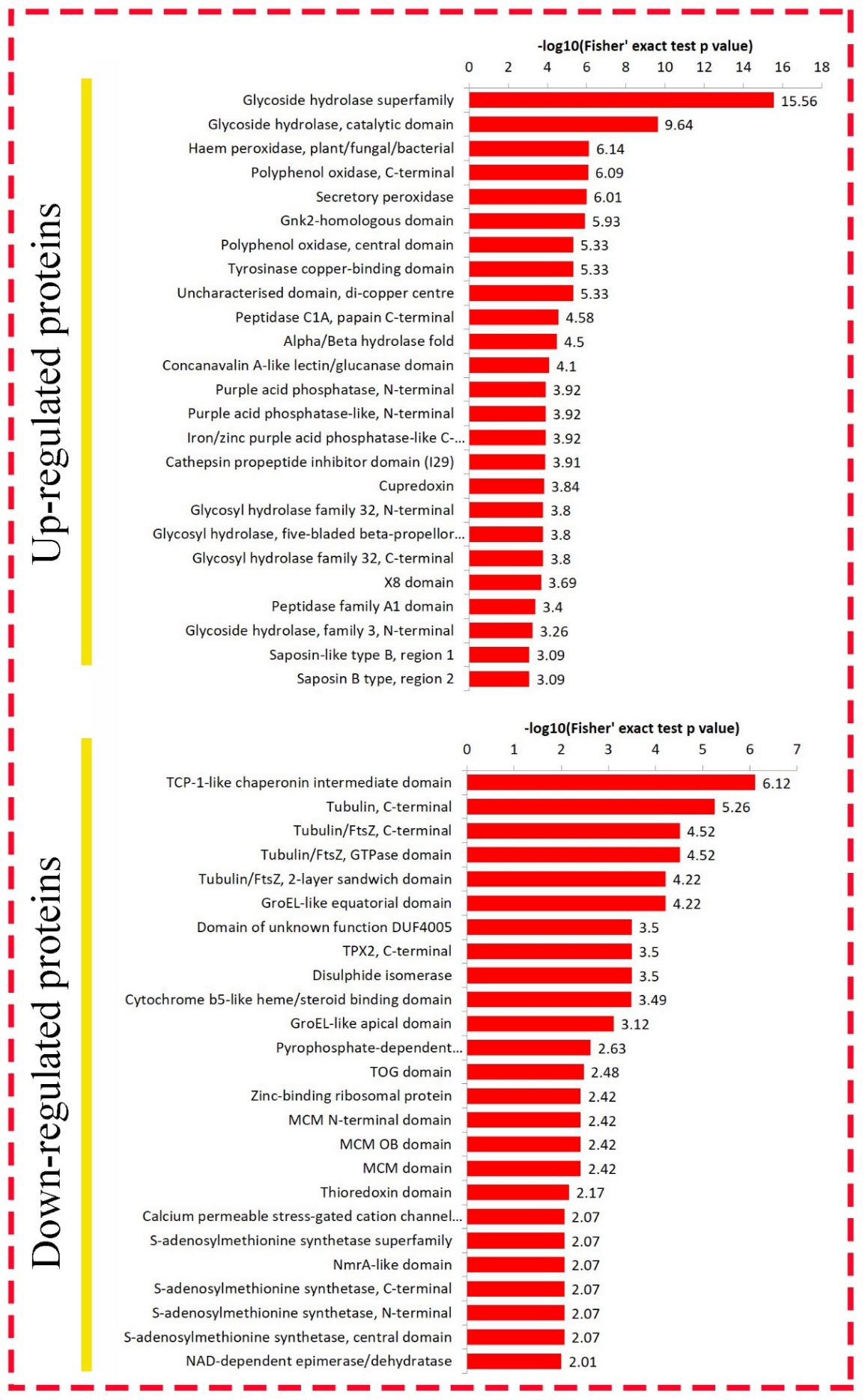


Figure.S3 Protein domain enrichment analysis of the differential accumulated proteins (DAPs) during the abscision process of carpopodium.
