# Supplementary material for: Comparative Proteomics Profiling Illuminates the Fruitlet Abscission Mechanism of Sweet Cherry as Induced by Embryo Abortion
Source: Int J Mol Sci. 2020 Feb 11;21(4):1200. doi: 10.3390/ijms21041200 (PMC7072775; doi:10.3390/ijms21041200)
Supplement: Supplementary file 1 [file ijms-21-01200-s001.zip › Supplementary Files-IJMS/Figure S1.doc]

Figure.S1 Variations in protein abundances between the abscissing carpopodium (CA) and non-abscissing carpopodium (CN). **(a)** The numbers of up- and down-regulated proteins in the abscissing carpopodium and non-abscissing carpopodium. **(b)** Gene ontology (GO) analysis of the up- and down-regulated proteins. All proteins were classified by GO terms based on their cellular component, molecular function, and biological process. Subcellular localization of up-regulated proteins **(c)** and down-regulated proteins **(d)**.
